# Renal Cell Cancer Diagnosed at Endoscopy

**DOI:** 10.1155/2012/360560

**Published:** 2012-11-12

**Authors:** Muhammed Hameed Thoufeeq, Nima Maleki, Naeem Jagirdar, Bjorn Rembacken, Jason Jennings

**Affiliations:** ^1^Gastroenterology, Leeds Teaching Hospitals, 50 Myrtle Way, Brough, Leeds HU15 1SR, UK; ^2^Pathology, Leeds Teaching Hospitals, Leeds, UK; ^3^Radiology, Leeds Teaching Hospitals, Leeds, UK

## Abstract

A 59-year-old lady was referred for an open-access endoscopy with a history of dyspepsia. The endoscopy showed a 5 mm sessile nodule in the fundus of the stomach. The histology report suggested that this represented a metastatic deposit from renal cell carcinoma (RCC). Following this, a computerised tomography (CT) of the abdomen showed an 18 × 15 cm RCC. Here we provide a short review on gastric metastases.

## 1. Case

A 59-year-old lady was referred for an open-access endoscopy with a history of dyspepsia.

She had a long standing history of reflux disease. Besides dyspepsia, she had a history of left lower back pain for 2 months. There were no urinary or bowel symptoms. She was prescribed Co-codamol and Celebrex. Celebrex made her dyspepsia worse hence she went to her general practitioner. She was a nonsmoker and did not drink much alcohol. Being very active, she lived with her husband whom she cared for. There was a family history of cancer; her father developed lung cancer at age 50 and her mother developed breast cancer at 80.

The blood results were as follows: haemoglobin of 11.7 g/dL (11.5–16.0), mean cell volume MCV 79 fL (78–100), neutrophil count of 3.99 × 10^9^/L (1.5–7.0), and platelets 357 × 10^9^/L (150–400). The biochemistry results were as follows: sodium 141 mmol/L (137–144), potassium 4.5 mmol/L (3.5–4.9), creatinine 80 *μ*mol/L (60–110), and urea 6.9 mmol/L (2.5–7.0).

**Figure 1:**
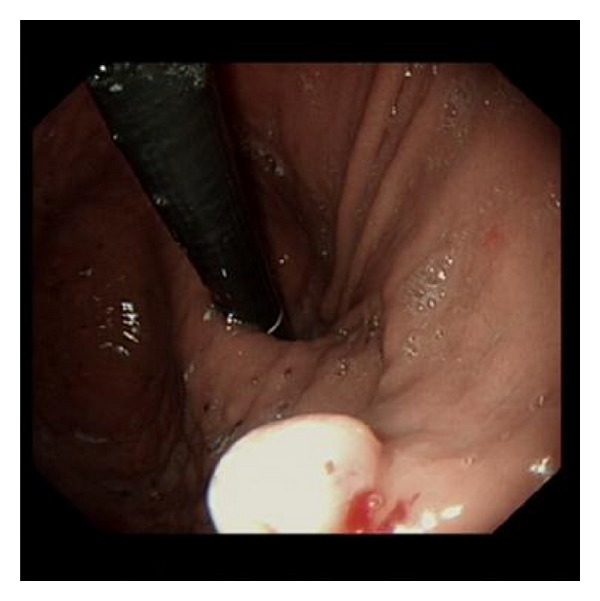
Oesophagogastroduodenoscopy (OGD) showing a gastric nodule.

 Endoscopy (Figure 1) showed a 5 mm sessile nodule in the fundus of the stomach. This nodule was sampled for histological analysis.

**Figure 2:**
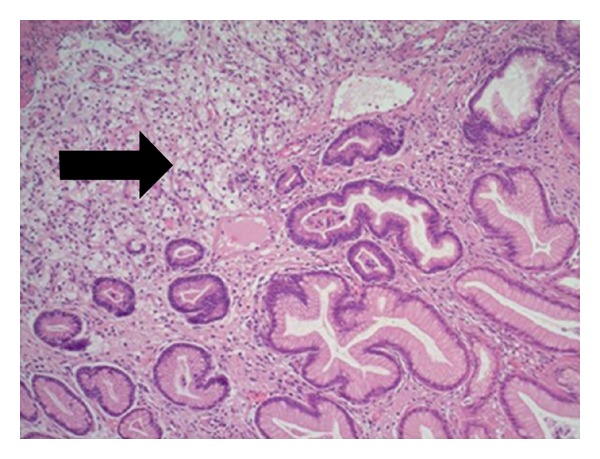
Microscopy of gastric nodule.

**Figure 3:**
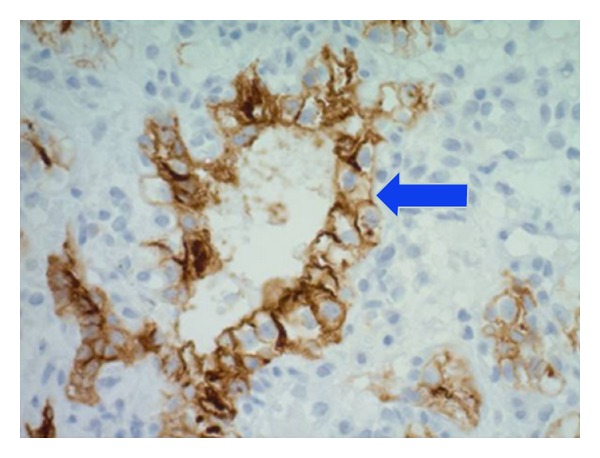
Immunostaining showing cancer cells staining positive for CD10.

The solitary sessile nodule in the fundus of the stomach was sampled.  Figure 2 shows microscopy of the nodule with sections of fundic gastric mucosa overlying a tumour in the lamina propria comprising large cells which exhibit clear cytoplasm (arrow).  Figure 3 shows cancer cells staining positive for CD10 on immunostaining (arrow). This nodule represents a secondary deposit from a primary renal cell carcinoma. 

**Figure 4:**
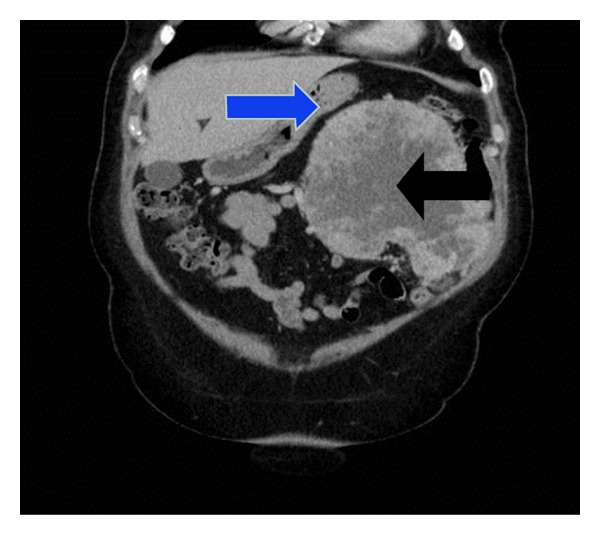
Computerised tomography (CT) scan of abdomen showing the renal mass (black arrow) close to the stomach (blue arrow).

Following this, a computerised tomography (CT) of the abdomen was arranged. This (Figure 4) showed an 18 × 15 cm tumour originating from the left kidney. The renal mass does not invade the stomach.

 At the time of writing up this paper she had developed cerebral metastases from her RCC. She had been started on Sunitinib, a multitargeted tyrosine kinase inhibitor by oncologists.

## 2. Discussion

Dyspepsia is a common indication for oesophagogastroduodenoscopy (OGD). About 40% of those endoscoped on presenting with dyspepsia have abnormal endoscopic findings with cancer usually being less than 1% [1]. Alarm symptoms in dyspepsia include weight loss, dysphagia, and anaemia which indicate a need for urgent OGD. However, they seem to have limited value in predicting cancer in dyspepsia [2]. About 25% of those presenting with upper gastrointestinal cancer do not have any alarming symptoms [3].

The differentials that were thought likely at the time of endoscopy were hyperplastic polyp, carcinoid, large xanthelasma, or a metastasis. (See Table 1).

Renal cell cancer (RCC) is known for its metastatic potential with up to a 1/3 rd of patients presenting with metastases. The presenting features of RCC include frank haematuria, flank pain, or abdominal mass. 

In general, metastases to stomach are very rare accounting for only 0.2–0.7% of gastric neoplasms [4]. The risk of metastasis in RCC is thought to be related to size of the tumour with risk being minimal if tumour is less than 3 cms [5].

Metastases to the GI Tract are usually secondary to melanoma, breast, and lung [6, 7]. In a case series of 8 cases with metastatic gastric tumours, 1 of them had a primary RCC [8]. The upper part of the stomach is most common site where metastases deposit in the stomach as seen in our case [9]. Renal cell carcinoma spreading to the stomach has been reported previously [10–12]. But they have all been in those who were known to have RCC already unlike our case. 

Metastases of RCC to other parts of the GI tract have also been reported, particularly to the small bowel [13, 14].

Endoscopically, gastric metastases usually appear like a submucosal tumour with smooth pattern having a colour blending with the surrounding mucosa with or without ulceration or resemble early or invasive gastric cancer as an ulcerated or a polypoid lesion [9, 15]. The gastric metastases could either present as solitary metastasis (65%) or multiple metastases (35%) [9]. Gastric metastases can present with dyspepsia like in our case or with bleeding. Only a small present (3.7%) of secondary gastric metastases present prior to their primary being diagnosed [9].

Sunitinib, a tyrosine kinase inhibitor has been found to prolong progression-free survival in patients with RCC with metastasis [16]. There is no definitive surgical or endoscopic treatment that has been shown to prolong survival. There is a case report in literature describing endoscopic mucosal resection of a mucosal secondary to the stomach which was found to be curative [17]. This patient had presented with secondaries to the stomach 3 years after radial nephrectomy for RCC.

## 3. Conclusions

Careful endoscopic examination should be carried in patients presenting with dyspepsia. Suspicious gastric nodules should be sampled for histological analysis. British Society of gastroenterology advices all gastric polypoid nodules except for fundal gastric polyps to be assessed histologically either by sampling or removal [18].

**What Is Already Known?**

 Metastases in the stomach are very rare.
 Renal cell carcinoma can spread to the stomach in someone with established disease.

**What Does This Case Add?**

 Gastric metastases can be the presenting feature of renal cell carcinoma.

## Figures and Tables

**Table 1:** 

Differentials for solitary gastric nodules
(1) Hyperplastic polyp
(2) Adenomatous polyp
(3) Inflammatory polyp
(4) Hamartomatous polyp
(5) Adenocarcinoma
(6) Gastric carcinoids
(7) Gastrointestinal stromal tumour (GIST)
(8) Lymphoma
(9) Xanthelasma
(10) Ectopic pancreas
(11) Fibroma and fibrolipoma
(12) Neurogenic and vascular tumours
(13) Metastatic deposit
